# Physical Activity, Air Pollution, and Mortality: A Systematic Review and Meta-analysis

**DOI:** 10.1186/s40798-025-00830-z

**Published:** 2025-04-07

**Authors:** Louise Martin, Hijrah Nasir, Reza Bagheri, Ukadike C. Ugbolue, Catherine Laporte, Julien S. Baker, Yaodong Gu, Marek Zak, Martine Duclos, Frédéric Dutheil

**Affiliations:** 1 General Medicine, Université Clermont Auvergne, Clermont-Ferrand, France; 2 Laboratory of the Metabolic Adaptations to Exercise Under Physiological and Pathological Conditions (AME2P), Chaire « Santé en Mouvement », Université Clermont Auvergne, Clermont-Ferrand, France; 3 Exercise Physiology Department, University of Isfahan, Isfahan, Iran; 4 Health and Life Sciences, Institute for Clinical Exercise & Health Science, University of the West of Scotland, University of Strathclyde, Glasgow, Scotland, UK; 5 Clermont Auvergne INP, CNRS, CHU Clermont-Ferrand, Institut Pascal, Université Clermont Auvergne, 63000 Clermont-Ferrand, France; 6 Centre for Health and Exercise Science Research, Hong Kong Baptist University, Kowloon Tong, Hong Kong; 7 Faculty of Sports Science, Ningbo University, Ningbo, Zhejiang China; 8 Collegium Medicum, Institute of Health Sciences, The Jan Kochanowski University, Kielce, Poland; 9 INRAe, CHU Clermont-Ferrand, Sport Medicine, Université Clermont Auvergne, Clermont-Ferrand, France; 10 LaPSCo, Physiological and Psychosocial Stress, University Hospital of Clermont–Ferrand, CHU Clermont–Ferrand, CNRS, Occupational and Environmental Medicine, Université Clermont Auvergne, WittyFit, 58 Rue Montalembert, 63000 Clermont–Ferrand, France

**Keywords:** Pollution, Mortality, Physical activity, Public health, Systemic review, Meta-analysis

## Abstract

**Background:**

As whether the positive effects of physical activity on mortality outweigh the negative effects of exposure to pollution is still under debate, we conducted a systematic review and meta-analysis on the risk of mortality for combined exposure to physical activity and air pollution.

**Methods:**

PubMed, Cochrane, Embase and ScienceDirect databases were searched for studies assessing the risk of mortality for combined exposure to physical activity and air pollution.

**Results:**

We included eight studies for a total of 1,417,945 individuals (mean 57.7 years old, 39% men) – 54,131 died. We confirmed that air pollution increased the risk of mortality by 36% (OR 1.36, 95CI 1.05–1.52), whereas physical activity in a non-polluted environment decreased the risk of mortality by 31% (OR 0.69, 95CI 0.42–0.95). Our meta-analysis demonstrated that combined exposure to physical activity and air pollution decreased the risk of mortality by 26% (OR 0.74, 95CI 0.63–0.85). This risk decreased whatever the level of physical activity: by 19% (OR 0.81, 95CI 0.69–0.93) for low, by 32% (OR 0.68, 95CI 0.44–0.93) for moderate, and by 30% (OR 0.70, 95CI 0.49–0.91) for high physical activity in air pollution.

**Conclusion:**

We confirmed that air pollution increased mortality by 36% in our meta-analysis. Despite the controversial benefit-risk, we demonstrated a reduction of mortality by 26% for combined exposure to physical activity and air pollution – nearly comparable to the reduction of mortality when practicing physical activity without air pollution (− 31%). However, the limited number of included studies precluded the demonstration of a dose–response relationship between levels of physical activity and air pollution, and reduction of mortality.

**Supplementary Information:**

The online version contains supplementary material available at 10.1186/s40798-025-00830-z.

## Highlights

- Air pollution is the cause of one in six deaths worldwide and is present in more than 99% of the world's territory.
- Physical activity helps reduce mortality.
- The risk-benefit ratio of physical activity in a polluted environment is unclear.
- We demonstrated a reduction of mortality for combined exposure to physical activity and air pollution which was nearly.
- Comparable to the reduction of mortality when practicing physical activity without air pollution.

**Supplementary Information:**

The online version contains supplementary material available at 10.1186/s40798-025-00830-z.

## Introduction

Pollution is responsible for one in six deaths worldwide [1], and is considered by the World Health Organization (WHO) as one of the main public issues facing the 21st century [2]. Pollution increases mortality in both the short and long term [3–6]. Another public health priority of the WHO is to promote physical activity [7]. People who do not engage in sufficient physical activity have a 20 to 30% increased risk of death [7]. The daily practice of 15 min of physical activity reduces the risk of all-cause mortality by 14% [8]. However, over 99% of the world population is living and breathing in air considered polluted [9], with 453 million people living in highly polluted megalopolises [10] – levels ten times higher than levels during the first global Covid lockdown have been measured in some cities [11–13]. There is an ongoing debate about the benefits of practicing physical activity in an air polluted environment [14], because physical activity may increase the absorption of air pollutants [15] as a result of elevated respiratory rate and minute ventilation [16]. Physical activity reduces inflammation-related proteins such as C-reactive protein (CRP) and interleukin-6 (IL-6), while pollution increases inflammation, highlighting a complex pathway in the long-term interaction between exercise and environmental factors [16]. Some studies recommended avoiding physical activity in polluted environments such as the roadside [17]. For example, physical activity in a polluted environment has been associated with an increased risk of cardio-respiratory or immune problems [18]; however, the impact on mortality is still debated, with pollution and physical activity having contradictory effects on mortality, and possibly interacting together [15, 19]. To our knowledge, no meta-analysis has assessed the effects on mortality of physical activity in an air polluted environment. Data on the possible dose–response effect of physical activity in air pollution on risk of mortality are also lacking. Moreover, while the effects of variables such as age, sex, body mass index, smoking, or alcohol on mortality in an air polluted environment or when practicing physical activity are well known [20–26], the effects of those variables on mortality when simultaneously practicing physical activity in air pollution are less well known.

Therefore, the main objective of this study was to conduct a systematic review of literature and a meta-analysis to determine the putative benefits on mortality of combined exposure to physical activity and air pollution. Secondary aims were to evaluate the dose–response effect of physical activity and air pollution, and to assess the influence of individual characteristics.

## Methods

### Literature Search

**Fig. 1:**
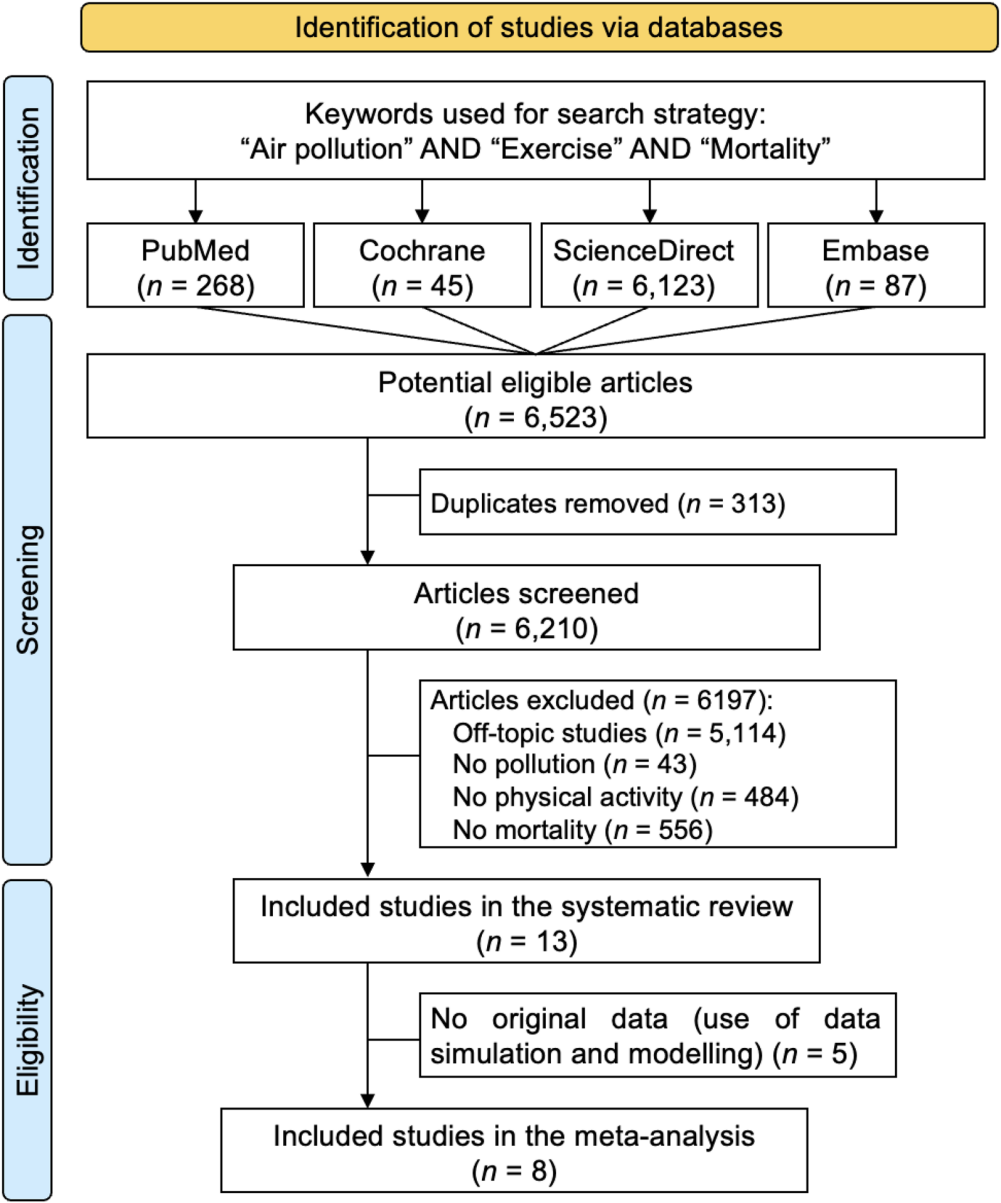
Search strategy. We followed the Preferred Reporting Items for Systematic Reviews and Meta-Analyses (PRISMA) guidelines for the search strategy

We reviewed all studies assessing the risk of mortality for combined exposure to physical activity and air pollution. The PubMed, Cochrane Library, Embase and ScienceDirect databases were searched until December 27th, 2023 with the following keywords: “mortality” and “pollution” and “physical activity” (details of the search strategy are available in Electronic supplementary material Table S1). The search was not limited to specific years and no language restrictions were applied. To be included in the systematic review, articles needed to describe our primary outcome i.e. mortality risk for combined exposure to physical activity and air pollution (Table 1). Then, articles using original data and describing either an odds ratio, a relative risk, or a hazard ratio, or giving data to calculate this risk, were included in the meta-analysis (Table 2). For example, studies using data simulation and modelling were included only in the systematic review, as well as physiological studies or if they were non-comparable cohort studies, but not in the meta-analysis. We also manually searched for any further studies using the reference lists from all included articles, and from reviews retrieved with our keywords. The search strategy is presented in Fig. 1. Two authors (Louise Martin and Hijrah Nasir) conducted all literature searches, collated, and reviewed the abstracts and based on the selection criteria, decided the suitability of the articles for inclusion. A third author (Frédéric Dutheil) was asked to review the eligible articles. We followed the guidelines outlined by Preferred Reporting Items for Systematic Reviews and Meta-Analyses (PRISMA).

**Table 1:** Characteristics of studies included in the systematic review but not in the meta-analysis

Study	Period	Country	Design	Risk	Physical activity	Pollution	Mortality	Result	Reason for non-inclusion in the meta-analysis
Coleman et al. 2022 [110]	1997–2014	USA	Prospective cohort	HR per 10 µg/m^3^ PM_2.5_ increase	HHS classifications: inactive (0 min per week of aerobic activity), insufficiently active (< 150 min), sufficiently active (150–300 min) and highly active (> 300 min)	PM_2.5_ By on-the-ground monitoring and satellite-based spatiotemporal models	All-cause Using ICD-10	Risk of mortality from pollution exposure increases despite physical activity	Unmatched risk
Giallouroso et al. 2020 [111]	2010–2016	Taiwan	Modeling study	RR	Active commuting (cycling or walking) based on UK national census estimations regarding daily commuting distance and Danish estimations regarding speed Then convert to MET	PM_2.5_ By on-the-ground monitoring from 2017 WHO database	All-cause (modelized data)	Avoiding walking and cycling on high air pollution days did not lead to reduce mortality risk	Modeling study
Pasqua et al. 2018 [112]	2016	USA	Modeling study	RR	30-min run, convert to MET/hour	PM_2.5_, PM_10_ By on-the-ground monitoring from 2016 WHO database	All-cause (modelized data)	In high air pollution: after 15 min of physical activity there are no more benefits and after 75 min there is adverse health effects	Modeling study
Tainio et al. 2016 [113]	2014	China	Modeling study	RR	Active commuting (cycling or walking) converts to MET/hour	PM_2.5_ By on-the-ground monitoring from 2014 WHO database	All-cause (modelized data)	Active travel in air pollution is beneficial in reducing the risk of mortality	Modeling study
Wong et al. 2007 [114]	1998	China	Retrospective cohort	ER per 10 μg/m3 air pollutants increase	Exercise or never-exercise (At least one time per month or zero)	NO_2_, SO_2_, PM_10_, O_3_ By on-the-ground monitoring from Environmental Protection Department and Hong Kong Observatory	All-cause and cardiorespiratory Using ICD-9	For people exposed to air pollution, physical activity is still beneficial in reducing the risk of mortality	Unmatched risk

ICD-9: International Classification of Diseases 9th Revision, ICD-10: International Classification of Diseases 10th Revision, HHS: United States Department of Health and Human Services, HR: hazard ratio, MET: Metabolically Equivalent of Task, RR: relative risk, PM_2.5_: particulate matter < 2.5 µm, PM_10_: particulate matter < 10 µm, WHO: World Health Organization

**Table 2:** Characteristics of studies included in the meta-analysis (articles using original data and describing either an odds ratio, a relative risk, or a hazard ratio, or provide data to calculate this risk)

Study	Country	Period	Design	Population				Mortality			Pollution			Groups		
				Total, n	Men, %	Age, years	Type	Type	Adjustment		Type	Measure by		Air pollution & no PA	PA & no pollution	PA in high air pollution
Andersen et al. 2015 [43]	Denmark	1993–2010	Prospective cohort	52,061	47.5	56.5 ± 4.3	General population	All-cause	Age, sex, marital status, education, income, occupational exposure, smoking, alcohol, nutrition, physical activity at work, NO_2_		N_O2_	Danish AirGIS dispersion modeling system		X	X	
Bo et al. 2022 [41]	Taiwan	2001–2016	Prospective cohort	384,124	48.7	39.2 ± 12.7	Local community	By cancer	Age, sex, education, occupational exposure, season, BMI, smoking, alcohol, nutrition, physical activity at work		PM_2.5_	Satellite-based spatiotemporal models		X		X
et al. 2020 [46]	USA	1988–2008	Prospective cohort	104,990	0	63.1 ± 8.9	Nurse	All-cause	Age, race, marital status, education of husband, income, home value, occupation of parents, registered nursing degree, retirement status, smoking, alcohol, nutrition, cancer, family history of myocardial infarction		PM_2.5_	Spatiotemporal prediction models			X	X
Guo et al. 2022 [44]	Taiwan	1994- 2019	Prospective cohort	384,130	50.4	41.6 ± 12.5	General population	By pneumonia	Age, sex, education, city, occupational exposure, year of enrolment, season, BMI, smoking, alcohol, nutrition, physical activity at work		PM_2.5_	Satellite-based spatiotemporal models		X		X
Ku et al. 2023 [47]	Taïwan	1994–2016	Prospective cohort	21,276	45.8	-	Targeted population	All-cause	Age, sex, marital status, education, household income, urbanization levels, BMI, smoking, alcohol, nutrition, Charlson Comorbidity Index, number of health checks		PM_2.5_	Satellite-based spatiotemporal models		X		X
Lin et al. 2021 [45]	China	1998–2008	Prospective cohort	76,176	40.7	51.2 ± 11.8	General population	All-cause	Age, sex, education, household income, urban or rural resident, geographic area, BMI, smoking, alcohol, nutrition, moderate-vigorous physical activity and sitting, HBP, pre-existing chronic conditions such as CVD, diabetes, dyslipidaemia, PM_2.5_		PM_2.5_	Satellite-based spatiotemporal models		X		X
Luo et al. 2023 [42]	UK	2006–2021	Prospective cohort	336,545	47.6	57 ± 7	General population	All-cause	Age, sex, education, recruitment center, deprivation index (unemployment, car/house ownership, household overcrowding), BMI, smoking, alcohol, nutrition, HPB		PM_2.5_	Regression model			X	X
Sun et al. 2018 [40]	China	1998–2011	Prospective cohort	58,643	34.3	71.9 ± 5.5	Targeted population	Cardiovascular and respiratory	Age, sex, education, financial expenditure, geographic area, BMI, smoking, smoking rate at district, alcohol, medication, pre-existing chronic conditions		PM_2.5_	Satellite-based spatiotemporal models			X	

BMI: body mass index, HBP: high blood pressure, PM_2.5_: particulate matter < 2.5 µm, CVD: cardiovascular disease

### Data Collection

The data collected included authors' names, publication year, study design, country, periods of studies, aims and outcomes of included articles, sample size, age, sex (percentage of males), risk of mortality (crude or adjusted, variables used for adjustment, type of risk – hazard ratio, odds ratio, relative risk), characteristics of pollution exposure (types, level), characteristics of physical activity (types, level, frequency) and characteristics of individuals (such as age, sex, education, body mass index (BMI), smoking, alcohol, high blood pressure).

### Quality of Assessment

**Fig. 2:**
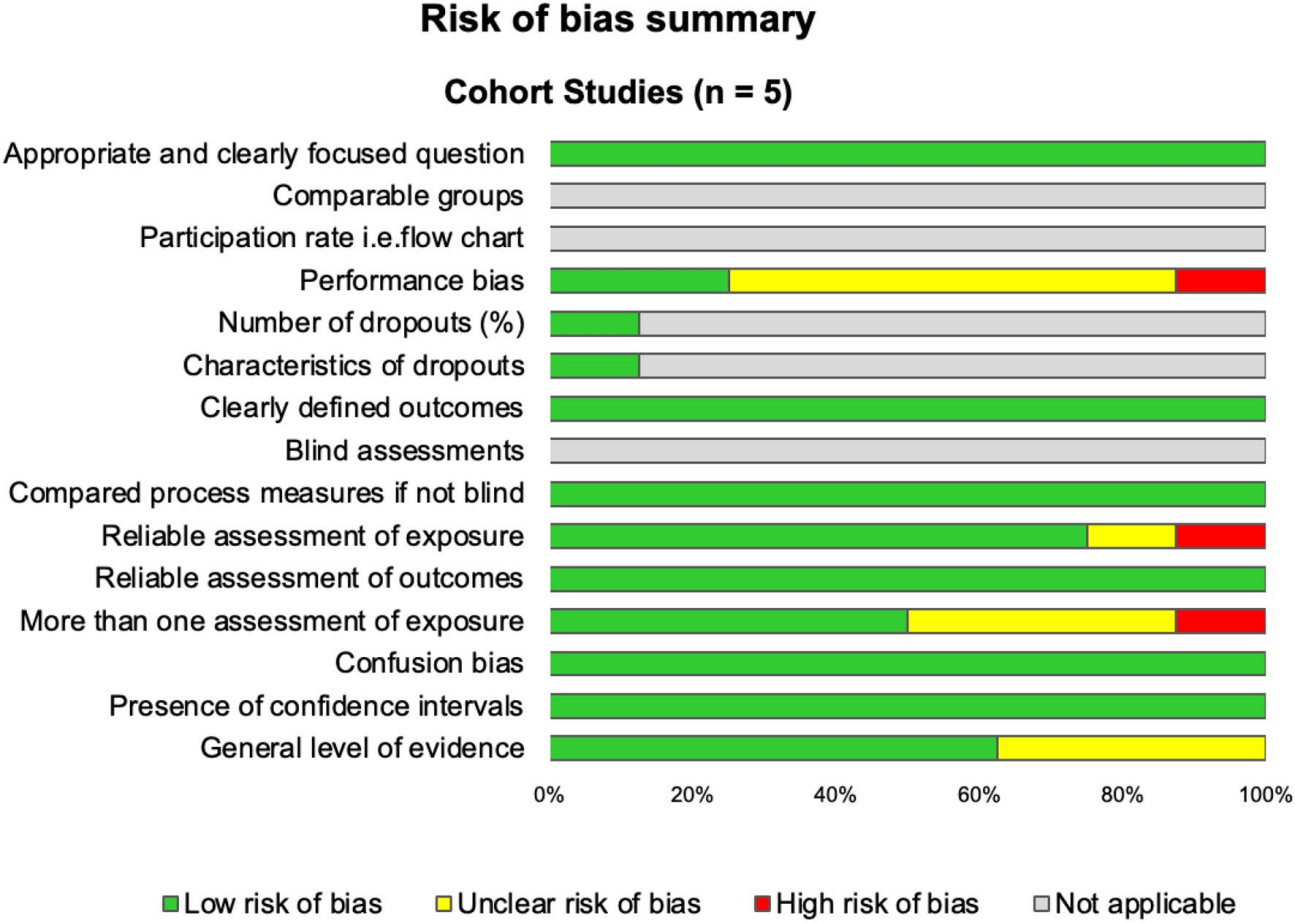
Risk of bias summary using the Scottish Intercollegiate Guidelines Network (SIGN) checklist. We used the Scottish Intercollegiate Guidelines Network (SIGN) grid for cohort studies that consist of 14 items, evaluating the main causes of bias through 4 possible answers (yes, no, can’t say or not applicable)

We used the Scottish Intercollegiate Guidelines Network (SIGN) grid for cohort studies that consists of 14 items, evaluating the main causes of bias through 4 possible answers (yes, no, can’t say or not applicable) [27] (Fig. 2). We used the Newcastle–Ottawa Scale (NOS) to assess the risk of bias of included articles [28]. Nine items contribute to evaluate three dimensions: selection, comparability, and outcome. One point was given for each item, i.e., a maximum score of 9 (Electronic supplementary material Fig. S1). The evidence synthesis for this review was conducted in accordance with the Grading of Recommendations Assessment, Development and Evaluation (GRADE) guidelines [29] (Electronic supplementary material Table S2).

### Statistical Considerations

**Fig. 3:**
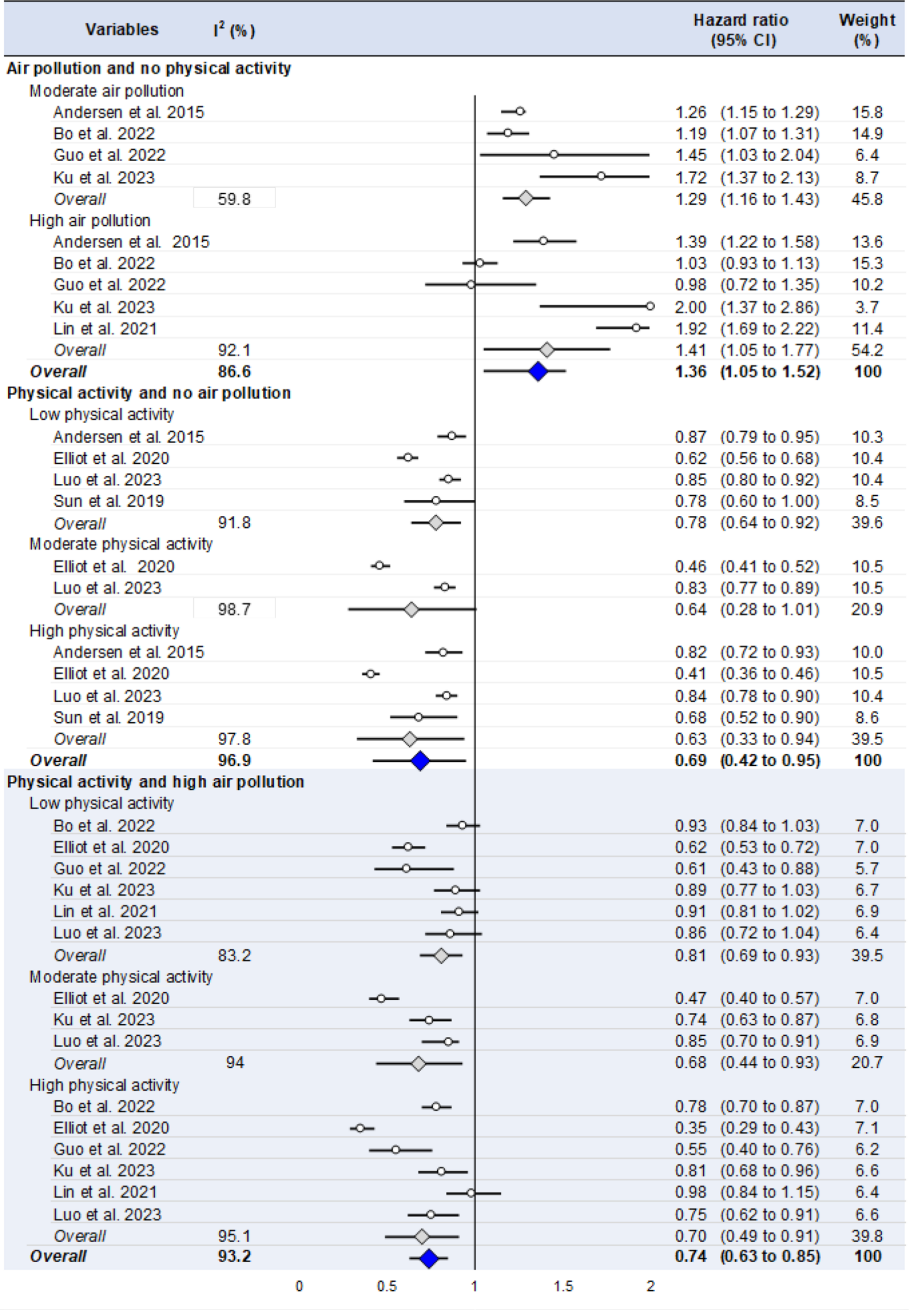
Summary of meta-analyses on the risk of mortality for (1) air pollution and no physical activity, (2) physical activity and no air pollution, (3) physical activity and high air pollution. Blue diamonds represent the overall risk of mortality after exposure to (1) air pollution and no physical activity, (2) physical activity and no air pollution, or (3) physical activity and high air pollution. Grey diamonds represent risk of mortality by level of exposure (low, moderate, high) for each type of exposure. Circles represent the risk of mortality for each included study. The length of each horizontal line around the circles /diamonds represents their 95% confidence interval (95CI). The black solid vertical line represents the null risk of mortality (with a value of 1). When horizontal lines cross the null vertical line, the risk of mortality is not significant. I-squared (%): percentage of heterogeneity between studies for each meta-analysis; Weight (%): Weight of each study for each meta-analysis

Statistical analysis was conducted using Stata software (v15, StataCorp, College Station, US) [30–33]. Baseline characteristics were summarized for each study sample and reported as mean (standard-deviation) for continuous variables and number (%) for categorical variables. Random effects meta-analyses (DerSimonian and Laird approach) were conducted when data could be pooled [34]. We conducted meta-analysis on the risk of mortality following 1) exposure to air pollution and no physical activity, 2) physical activity and no air pollution, and 3) both physical activity and air pollution [35]. We stratified these meta-analyses on the level of pollution (moderate and high air pollution) and physical activity (low, moderate, and high physical activity) given by studies (Fig. 3). The risk of mortality was centered at 1 when not differing between the exposure (air pollution, or physical activity, or both physical activity and air pollution) and the absence of exposure. A risk > 1 reflects an increased risk of mortality, while a risk < 1 reflects a reduced risk. Heterogeneity between risks of mortality in each study was evaluated by examining forest plots, 95% confidence intervals (95CI) and I-squared (I^2^). I^2^ measures between-study heterogeneity: 0 < I^2^ < 25% reflects a low heterogeneity, 25 < I^2^ < 50% a modest heterogeneity, and 50 < I^2^ < 100% a high heterogeneity. For rigor, funnel plots (metafunnels) of these meta-analyses were also used to search for potential publication bias. We verified the strength of our results by repeating the aforementioned meta-analyses after exclusion of outliers i.e. after exclusion of studies not evenly distributed around the base of the funnel [36], as well as sensitivity analyses based on the type of risks (hazard ratio, odds ratio, or relative risk). Moreover, when a study reported simultaneously both crude and adjusted risks, we computed two models (a model using the most adjusted risks listed in included articles, and a model using only crude or less adjusted risks). When possible (sufficient sample size), meta-regressions were proposed to search for relationships between the risk of mortality and levels of air pollution, levels of physical activity, and levels of both physical activity and air pollution, as well as sociodemographic (age, sex, education) and clinically relevant parameters (BMI, smoking, alcohol, high blood pressure) (Electronic supplementary material Fig. S2). Results were expressed as regression coefficients (Coeffs) and 95% CIs. P values less than 0.05 were considered statistically significant.

## Results

An initial search produced a possible list of 6523 articles. Removal of duplicates and application of the selection criteria reduced the number of articles reporting the risk of mortality for combined exposure to physical activity and air pollution to 13 articles in the systematic review and to 8 articles for the meta-analysis (Fig. 1). All articles were written in English. The main characteristics of studies included only in the systematic review are presented in Table 1, and characteristics of studies included in the meta-analysis are summarized below and in Table 2.

### Risk of Bias and Certainty of Evidence

The assessment of the quality of the eight included studies was performed using the NOS and the SIGN checklist to assess the risk of bias and GRADE to assess the certainty of evidence [28, 37–39]. According to the NOS, the study quality was high for three studies [40–42], moderate for three of them [43–45] and low for two of them [46, 47]. The SIGN checklist showed a low risk of bias for five studies [40, 41, 43, 44, 47] and an unclear risk of bias for three studies [42, 45, 46] (Fig. 2). All of them mentioned ethics approval [40–47]. The certainty of the evidence (GRADE) was rated as high for our main outcome and moderate for most secondary outcomes (Electronic supplementary material Table S2).

### Aims and Outcomes of Included Studies

The main objective of included studies was to evaluate the risk of mortality for combined exposure to physical activity and air pollution (primary outcome) [40–47]. Five studies assessed all-cause mortality [42, 43, 45–47], one study focused on cardiovascular and respiratory mortality [40], one study focused on mortality by cancer [41] and one only reported mortality by pneumonia [44].

### Study Design

Our eight studies were prospective cohorts, conducted between 1988 and 2016. Follow-up ranged from 10 [45] to 25 years [44]. Studies were located in Asia (China [45] and Taiwan [40, 41, 44, 47]), Europe (Denmark [43] and UK [42]) and North America (USA [46]).

### Recruitment, Inclusion, and Exclusion Criteria

Four studies recruited participants from general population via extended cohorts [42–45], three recruited targeted populations (patients over 65 years old [40], patient over 60 years old [47] and nurses [46]), and the other recruited from the local community [41]. The only inclusion criterion was age in five studies: over 18 [41, 44], between 50 and 64 [43], over 60 years old [47] and over 65 years old [40].

Seven studies excluded participants due to missing address or satellite data, making it impossible to determine the particulate matter < 2.5 μm in diameter (PM_2.5_) exposure [40–44, 46, 47]. Seven studies excluded participants with missing information on the physical activity [40–42, 44, 46, 47] or on the commuting [45]. Six studies excluded for missing covariates [40–44, 47]. Four studies excluded participants for having pre-existing chronic conditions, such as cardiovascular disease (CVD) [45, 46], cancer [43] or mood disorders and type 2 diabetes [42]. Two excluded the participants who were seen once [45, 47]. One study excluded unemployed and retired participants [45].

### Population

*Sample size* ranged from 21,276 [43] to 384,130 [44]. We included 1,417,945 individuals in total, of which 54,131 (3.82%) died. The prevalence of death ranged from 0.15 [44] to 16.63 [47] %.

*Age* was reported in all studies. The mean age was 57.7 years old (95% CI 49.9 to 65.6 years old), ranging from 39.2 ± 12.7 [41] to 71.9 ± 5.5 [40] years.

*Sex* was also reported in all studies. The mean proportion of men was 39% (16 to 63%), with proportion ranging from 0.0 [46] to 50.4% [44].

*Education* was reported in all studies except one [40]. The mean proportion of people with education beyond high school was 54% (30–78%), ranging from 12.3 [45] to 100% [46].

*BMI* was reported in all studies. Five studies reported means. The mean BMI was 23.9 kg/m^2^ (20.3–27.6 kg/m^2^), ranging from 23.0 ± 3.7 [41] to 26.6 ± 2.8 [42] kg/m^2^. Two studies reported the proportions of normal BMI, overweight and obese people [42, 46], and one study reported proportions of individuals with < 21, 21–26, > 26 kg/m^2^ [40].

*Smoking status* was reported in all studies for proportions of current and former smokers, except two that did not report former smoking [42, 45]. The mean proportion of current smokers was 21% (13–30%), ranging from 4.4 [40] to 44.7% [42]; the mean proportion of former smokers was 17% (11–24%), ranging from 5.7 [41] to 43% [46].

*Alcohol consumption* was reported in all studies except one [40]. The percentage of current or former consumers was 41% (12 to 69%), ranging from 14.1 [41] to 97.8% [43].

*High blood pressure (HBP)* was reported in four studies [40, 43, 45, 46]. The percentage of people with HBP was 32% (19–44%), ranging from 15.3 [43] to 43% [46].

*Other descriptive variables* were nutrition [41–47], marital status [43, 46, 47], level of NO_2_ [43], level of PM_2.5_ [45], risk occupation [43], physical activity at work [41, 43, 44], income [43, 46], occupational exposure [41, 44], season [41], race [42, 46], cancer [46], family history of myocardial infarction [46], home value [46], occupation of parents [46], education of husband [46], registered nurse degree [46], retirement status [46], urban or rural resident [45, 47], geographic area [40, 45], household income [45, 47], deprivation [42] moderate-vigorous physical activity and sitting [45], pre-existing conditions [40, 45, 47], medication [40], financial expenditure [40] and smoking rate at the district level [40].

### Evaluation of Mortality

Five studies retrieved mortality from national death registries: the Danish Register of Causes of Death [43], the National Death Index of the USA [46], the National Death Registry of the UK [42], the National Death Registry maintained by the Ministry of Health and Welfare of Taiwan [41, 44, 47] and the death registration in the Department of Health of Hong Kong [40]. One study [45] asked the proxies of participants to advise death information, then they validated the diagnosis by checking hospital records or death certificates. To classify the cause of death, seven studies used the International Classification of Diseases (ICD): the 9th revision [46], the 10th revision [40, 42, 43, 45] and the 9th and 10th revision [41, 44]. While the majority of the studies assessed all-cause mortality as the primary outcome [42, 43, 45–47], some employed more specific mortality such as by cancer [41] or by pneumonia [44] or cardiovascular and respiratory mortality [40].

### Evaluation of Air Pollution

Air pollution was assessed using a spatio-temporal model based on the residential address of individuals and based on indirect indicators of air pollution measured in the atmosphere by satellite [40, 41, 44, 45] or on-the-ground monitoring of air pollutants [42, 46, 47], or statistical modelling [43]. Indirect indicators of PM_2.5_ concentration in the troposphere were assessed using Aerosol Optical Depth (AOD) data from the National Aeronautics and Space Administration (NASA) [40, 41, 44, 45], with an algorithm permitting a 1 × 1 km resolution – MODerate resolution Imaging Spectroradiometer (MODIS) [40, 41, 44] and Multi-Angle Implementation of Atmospheric Correction (MAIAC) [45]. On-the-ground monitoring of air pollutants was achieved by the Environmental Protection Agency’s Air Quality System in the USA [46] and by the European Study of Cohorts of Air Pollution Effects (ESCAPE) [42] for PM_2.5_, and by the Environmental Protection Administration in Taiwan for several pollutants PM_2.5_, NO_2_, SO_2_, CO, and O_3_ [47]. One study assessed levels of air pollution using statistical modelling of NO_2_ concentration using the Danish Air Geographic Information System (AirGIS), that takes into account traffic information and previously validated against on-the-ground monitors [43]. All studies defined groups of exposure to air pollution based on only one air pollutant: PM_2.5_ in seven studies [40–42, 44–47] and NO_2_ in one study [43]. The level of air pollution used for statistical analyses was the average level of air pollution the two years preceding each medical examination in three studies [41, 44, 46], the last year preceding each medical examination in one study [47], the last year of follow-up in one study [42], and over the entire follow-up in three studies [40, 43, 45]. Levels of pollution were classified into two categories in three studies (PM_2.5_ below or above 61 [45], 35.3 [40] or 9.5 µg/m^3^ [42]), into three categories in four studies (NO_2_ < 15.1, 15.1 < NO_2_ < 23.9, NO_2_ > 23.9 µg/m^3^ [43]; PM_2.5_ < 22.2, 22.2 < PM_2.5_ < 25.9, PM_2.5_ > 25.9 [41, 44]; PM_2.5_ < 20, 20 < PM_2.5_ < 25, PM_2.5_ > 25 [47]), and into five categories in one study (PM_2.5_ < 10.7, 10.7 < PM_2.5_ < 12.4, 12.4 < PM_2.5_ < 14.3, 14.3 < PM_2.5_ < 25.9, PM_2.5_ > 25.9) [46]. Data could be pooled only in studies that homogeneously reported their results (Table 1).

### Evaluation of Physical Activity

All studies assessed physical activity using questionnaires: self-administered [41–44, 46, 47] or by trained staff [40, 45]. Only two studies used a validated questionnaire: the International Physical Activity Questionnaire (IPAQ) [42] and the European Prospective Investigation into Cancer and Nutrition (EPIC) physical activity questionnaire [43]. Except for one study that collected only the commuting mode [45], all studies assessed the type, duration, and intensity of physical activity. However, the type of physical activity retrieved differed between studies: walking [40–43, 45–47], cycling [40, 43, 45, 46], running or jogging [40, 41, 46, 47], swimming [40, 46, 47], tennis, squash or racquetball [46], calisthenics [46], weight training [46], yoga [46], stretching exercise [40], traditional Chinese exercise [40] and other aerobic activities [46] or other types not mentioned [40, 47] or sports without specification [43]. The assessments of physical activity used for statistical analyses were the initial assessment in three studies [40, 42, 45], the last update of physical activity in three studies [41, 43, 46], or the averaged physical activity over the follow-up – with participants completing a mean of two assessments [44, 47]. Six studies [40–42, 44, 46, 47] then calculated each participant’s metabolic equivalent of task (MET) hours per week. Three studies used MET-h/week to categorize participants into three groups: inactive (< 1 MET-h/week), moderate (1–8.75), and high (> 8.75) activity groups in two studies [41, 44]; < 1, 1–21, and > 21.0 in one study [40]. Three studies also used MET-h/week but categorized four groups based on quartiles: < 3.7, 3.7–10.9, 10.9–24.4, and > 24.4 [46]; < 1, 1–7.5, 7.5–15, and > 15 [47]; < 2, 2–8, 8–20, 20–94 [42]. One study defined three groups based on cycling frequency (no cycling, 0.5–4 h/week, < 4 h/week) [43], and one based on type of physical activity: none (inactive), walking, and cycling [45]. Similarly to air pollution, only results from some studies could be pooled (Table 1).

### Meta-analysis of the Risk of Mortality

*People exposed to air pollution, without physical activity*, have a 36% increased risk of mortality (OR 1.36, 95CI 1.19–1.52) compared to those not exposed to air pollution. Stratification by level of air pollution showed a 29% increased risk of mortality (OR 1.29, 95CI 1.16–1.43) for people exposed to a moderate level of air pollution, and a 41% increased risk (OR 1.41, 95CI 1.05–1.77) for people exposed to a high level of air pollution.

*People practicing physical activity and not exposed to air pollution* have a 31% decreased risk of mortality (OR 0.69, 95CI 0.42–0.95) compared to those who do not participate in a physical activity. Stratification by level of physical activity showed a 22% decreased risk of mortality (OR 0.78, 95CI 0.64–0.94) for people with a low level of physical activity, a close-to-significant 36% decreased risk (OR 0.64, 95CI 0.28–1.01) for those with a moderate level of physical activity, and a 27% decreased risk (OR 0.63, 95CI 0.33–0.94) for those with a high level of physical activity.

*People practicing physical activity and exposed to high level of air pollution* have a 26% decreased risk of mortality (OR 0.74, 95CI 0.63–0.85) compared to those not practicing any physical activity. Stratification by level of physical activity showed a 19% decreased risk of mortality (OR 0.81, 95CI 0.69–0.93) for people with a low level of physical activity, a 32% decreased risk (OR 0.68, 95CI 0.44–0.93) for those having a moderate level of physical activity, and a 30% decreased risk (OR 0.70, 95CI 0.49–0.91) for those having a high level of physical activity (Fig. 3).

#### Meta-regressions

Meta-regression did not show a dose–response relationship between levels of pollution without physical activity (Coeff 0.02, 95CI − 0.57 to 0.62, p = 0.94 for high vs low pollution), between levels of physical activity without air pollution (Coeff − 0.09, 95CI -0.41 to 0.23, p = 0.52 for high vs low physical activity), as well as between levels of physical activity and high air pollution (Coeff − 0.11, 95CI − 0.35 to 0.13, *p* = 0.34 for high vs low physical activity). Women have a higher risk of mortality than men when exposed to air pollution without physical activity (Coeff 1.01, 95CI 0.48 to 1.54, per 10%-women). Benefits of physical activity on mortality are more pronounced for women, both for physical activity without air pollution (Coeff − 0.07, 95CI -0.10 to -0.04, per 10%-women) or with air pollution (Coeff − 0.06, 95CI -0.12 to -0.01, per 10%-women). The risk of mortality is reduced for those with a level of education higher than high school whatever the group (Coeff − 0.08, 95CI − 0.15 to − 0.01 for air pollution without physical activity; Coeff − 0.07, 95CI − 0.12 to − 0.01 for physical activity without air pollution; and Coeff − 0.04, 95CI − 0.07 to − 0.02 for physical activity and high air pollution). Current smokers are at higher risk of mortality when practicing physical activity without air pollution (Coeff 0.07, 95CI 0.00–0.14, *p* = 0.050), as well as a tendency for those consuming alcohol (Coeff 0.06, 95CI − 0.00 to 0.13, *p* = 0.053), while former smokers exposed to air pollution tended to benefit more from physical activity (Coeff − 0.08, 95CI − 0.16 to 0.00, *p* = 0.055). People with high blood pressure have a greater decrease in the risk of mortality when they practice physical activity without air pollution (Coeff − 0.11, 95CI − 0.22 to − 0.01, *p* = 0.043), and tended to have a greater decrease in the risk of mortality when they practice physical activity while being exposed to air pollution (Coeff − 0.47, 95CI − 1.11 to 0.17, *p* = 0.089). The meta-regressions did not show any other influencing variables on the risk of mortality. Considering the limited number of studies, results of meta-regressions are available in electronic supplementary material Figure S2.

#### Sensitivity Analyses

Heterogeneity for the overall results of all aforementioned meta-analysis was very high (> 85%). Metafunnels (Electronic supplementary material Figure S3) confirmed the high heterogeneity with all studies outside the base of the funnel, precluding further sensitivity analyses. Similarly, all the studies reported only adjusted risks, and thus sensitivity analyses depending on adjusting factors were deemed impossible.

## Discussion

The main findings were that, for people exposed to air pollution, physical activity was still beneficial in decreasing mortality. For those people, physical activity seemed more beneficial on mortality in women and tended to be more beneficial in former smokers and in people with high blood pressure.

### Physical Activity and Air Pollution

An ongoing debate is the benefit-risk ratio of practicing physical activity in an air polluted environment [15, 19]. Our meta-analysis demonstrated that combined exposure to physical activity and air pollution is still beneficial in reducing the risk of mortality. In the literature, exposure to air pollution increases the risk of both short- and long-term mortality [48, 49]. This risk concerns all-cause mortality, or more specific mortality such as by lung cancer or cardiovascular or respiratory mortality [50, 51]. This increased risk of mortality has been shown both for acute or chronic air pollution exposure [48, 49, 52–55], household [56] or environmental air pollution, and for various pollutants – NO_2_, SO_2_, CO, O_3_, particulate matter < 10 μm in diameter (PM_10_) and PM_2.5_ [50, 52, 55, 57]. Besides the increased risk of mortality, air pollution has also a wide range of side effects, from cardiorespiratory disease [58] to cancer [59], and also birth defects [60–63]. Physical activity can reduce the risk of all-cause, cancer, cardiovascular and respiratory mortality [64–68], particularly for individuals who are usually sedentary [64]. Even the lowest level of physical activity is beneficial [66]. Furthermore, physical activity also showed long-term health benefits to prevent a wide range of diseases, from mental to cardiometabolic disorders [69–72]. Practicing physical activity in a polluted environment can lead to changes in physiological parameters, including high systolic blood pressure, pulmonary inflammation, and increased systemic markers of inflammation or immunomodulatory protein [15, 18]. In the literature, the risk of mortality does not appear to be influenced by the type of physical activity [65]. Unfortunately, the studies included in our meta-analysis did not assess the type of physical activity and only assessed levels of physical activity. In our meta-regression in the group exposed to high air pollution, we did not find a dose–response effect on mortality based on the level of physical activity. However, in the literature, moderate physical activity seemed to provide greater protection in a highly polluted environment, while intense physical activity seemed to have greater benefits in a moderately polluted environment [70]. There is a lack of evidence that facemasks decrease exposure to air pollution [73], but they have no influence on physical performance and a minimal impact on physiological variables [74]. Even though climate change and air pollution are an ongoing priority, this should not discourage people to exercise, with or without a facemask.

### Factors Influencing Mortality

Although we did not show any influence of age or BMI on mortality for air pollution without physical activity, physical activity without air pollution, and exposure to both physical activity and high air pollution, some relationships were demonstrated in the literature. The increased risk of mortality from exposure to pollution in older individuals may be linked with a longer exposure to pollution and a reduced ability to react to the stress of air pollution [75, 76], while the increased risk in the obese may be linked with their likelihood of having cardiorespiratory diseases that can be worsened by air pollution [77, 78]. Interestingly, physical activity decreases mortality risk regardless of age [79, 80] and BMI [81]. To our knowledge, no study has assessed the influence of age or BMI on the risk of mortality for combined exposure to physical activity and air pollution. As we did not show any relationship, physical activity may also be beneficial for the elderly, as well as for overweight or obese individuals despite air pollution. In line with the literature [76, 82], we showed that women are more at risk of mortality when exposed to air pollution, compared to men. Many pollutants are endocrine disruptors that affect women more; women’s airways are also more likely reactive due to their smaller caliber [83]. Conversely, we showed that women also seem to be more protected than men when practicing physical activity, whether in a non- or highly-polluted environment. In the literature, it has already been shown that physical activity has more benefits on mortality in women, but studies did not control for air pollution [79, 84, 85]. Physical activity may be more beneficial in women because they are less likely to take part in extreme high-risk sports [86–88], and they may also be protected against the cardiovascular risk [89, 90]. In all groups of our meta-analysis, the risk of mortality decreased with education, putatively because of less risky behaviors, such as smoking or drinking alcohol [91]. Such a protective effect of education was only reported in some studies for exposure to air pollution [76, 92], possibly because of reduced access to healthy food or medical care for pre-existing diseases [93]. Although we did not show an effect of smoking or alcohol on mortality when exposed to air pollution or for combined exposure to physical activity and air pollution, physical activity may increase the risk. In the literature, we did not find studies specifically assessing the effect of smoking or alcohol intake on mortality for combined exposure to physical activity and air pollution, but smokers and persons with an alcohol use disorder decrease their risk of mortality when they engage in physical activity [94–98], while acute exercise in non-trained individuals at high cardiovascular risk – such as smokers or persons with an alcohol use disorder – may increase the risk of mortality [99]. Exposure to air pollution also increases this risk in smokers [100, 101]. The pulmonary system may be more sensitive to air pollutants because of the hyper-reactivity caused by smoking [102]. In our meta-analysis, for people exposed to air pollution, physical activity may have been more beneficial on the risk of mortality for former smokers. Few studies focused on former smokers, but their mortality risk decreased with physical activity [96, 103] while it increased with air pollution [104]. In our meta-regression, practicing a physical activity in a non-polluted environment is beneficial in decreasing the risk of mortality for those who have high blood pressure, in line with the literature [105]. Other traditional factors that may influence the risk of mortality include chronic disease. People suffering from respiratory insufficiency or asthma who take part in physical activity benefit from a reduced risk of mortality, like the general population [106] but they are also more sensitive to the deleterious effects of air pollution [107–109]. However, limited data precluded such analysis in our study.

### Limitations and Strengths

Our meta-analysis has several limitations. We included only eight studies, but we have a very large sample size of 1.5 million individuals giving a high degree of confidence in the results of our meta-analysis. However, metaregressions may be interpreted with caution, and would benefit from a meta-analysis of individual data. Our meta-analysis also inherits the bias of individual included studies, such as their study design. However, study designs can only be observational in this case, and no included studies were cross-sectional. All studies had a long follow-up, between 10 [45] and 25 years [44], in favor of the robustness of our findings. This also allows us to better understand the long-term implications of combined exposure to physical activity and air pollution. Differences in health practices across the world may also influence our outcome (mortality). Moreover, studies used different times of assessment and approaches to assess both our two main variables (physical activity and air pollution) i.e. there was a bias of exposure assessment. Most studies did not use a validated standardized questionnaire, and some used a self-questionnaire without supervision. This heterogeneity in assessment methods introduced a source of variability which may have affected comparability between studies. Similarly, assessment of air pollution varied in the choice of air pollutants and in the levels used for classification. All studies tested only one or few pollutants, whereas air pollutants are often multiple. There was also heterogeneity between studies in the outcome i.e. mortality that was all-cause mortality or from specific causes in some studies [40, 41, 44]. Lastly, all studies were published over the last five years except one in 2015, precluding analyses of a time effect. Since in the last few years both temperature and air pollution have increased at a faster pace than in the past, it would have been of particular interest to analyze whether there was a difference between the earlier and more recent studies. Lastly, all risks were adjusted, precluding sensitivity analyses i.e. whether some factors modified the risk – for example as physical activity affects BMI and consequently affects mortality, BMI can act as a mediator; having several risks depending on adjusted factors may have strengthen our results. Despite these challenges, our meta-analysis contributes significantly to our understanding of the benefits on mortality of physical activity despite exposure to air pollution.

## Conclusion

We confirmed that air pollution increased mortality by 36% in our meta-analysis. Despite the controversial benefit-risk, we demonstrated a reduction of mortality by 26% for combined exposure to physical activity and air pollution – nearly comparable to the reduction of mortality when practicing physical activity without air pollution (− 31%). However, the limited number of included studies precluded the demonstration of a dose–response relationship between levels of physical activity and air pollution, and reduction of mortality. Even if climate change and air pollution are an ongoing priority, it should not discourage people to exercise.

## Supplementary Information

Additional file 1.

Additional file 2.

Additional file 3.

Additional file 4.

Additional file 5.

## Data Availability

All relevant data are within the paper.
