# Supplementary material for: Physical Activity, Air Pollution, and Mortality: A Systematic Review and Meta-analysis
Source: Sports Med Open. 2025 Apr 7;11:35. doi: 10.1186/s40798-025-00830-z (PMC11977067; doi:10.1186/s40798-025-00830-z)
Supplement: Supplementary file 1 — Additional file 1. [file 40798_2025_830_MOESM1_ESM.docx]

**Electronic supplementary material Fig. S1** Details for evaluation of quality of included studies (using the SIGN and NOS)

Details of quality of included studies using the SIGN checklist

The NOS Checklist


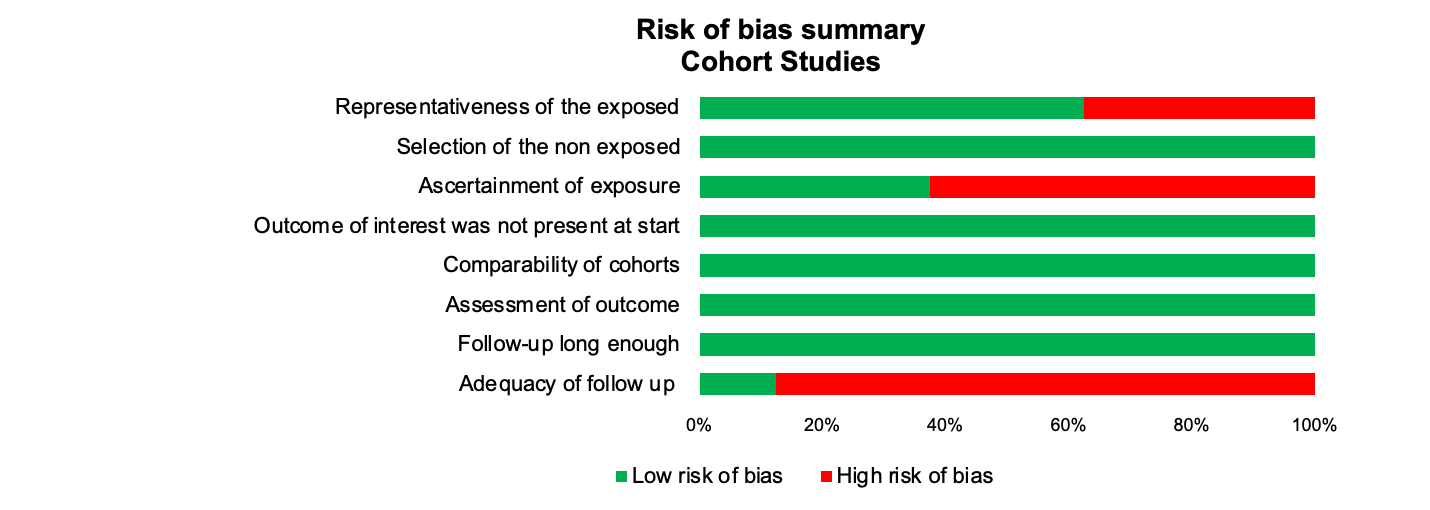


Details of quality of included studies using the NOS checklist
