# Supplementary material for: Physical Activity, Air Pollution, and Mortality: A Systematic Review and Meta-analysis
Source: Sports Med Open. 2025 Apr 7;11:35. doi: 10.1186/s40798-025-00830-z (PMC11977067; doi:10.1186/s40798-025-00830-z)
Supplement: Supplementary file 2 — Additional file 2. [file 40798_2025_830_MOESM2_ESM.docx]

### Electronic supplementary material Fig. S2 Metaregressions i.e. variables influencing the risk of mortality, depending on groups: 1) air pollution and no physical activity, 2) physical activity and no pollution, 3) physical activity in air pollution

The effect of each variable on the risk of mortality is represented by a dot on a horizontal line in the forest-plot. The dots represent the coefficient for each variable, and the length of each line around the dots represent their 95% confidence interval (95CI). The black solid vertical line represents the null estimate (with a value of 0). Horizontal lines that cross the null vertical line represent non-significant variables on the risk of mortality
