# Supplementary material for: Physical Activity, Air Pollution, and Mortality: A Systematic Review and Meta-analysis
Source: Sports Med Open. 2025 Apr 7;11:35. doi: 10.1186/s40798-025-00830-z (PMC11977067; doi:10.1186/s40798-025-00830-z)

**Electronic supplementary material Fig. S3** Metafunnels for each meta-analysis: air pollution and no physical activity, physical activity and no pollution, physical activity in air pollution

Each circle represents a single study, with its corresponding effect size (x axis) and its associated standard error of the effect estimate (y-axis). Large high-powered studies are placed towards the top, and smaller low-powered studies towards the bottom. The plot should ideally resemble a pyramid or inverted funnel, with scatter due to sampling variation. Studies outside funnel plot are likely to present bias [110]

**Air pollution and no physical activity**


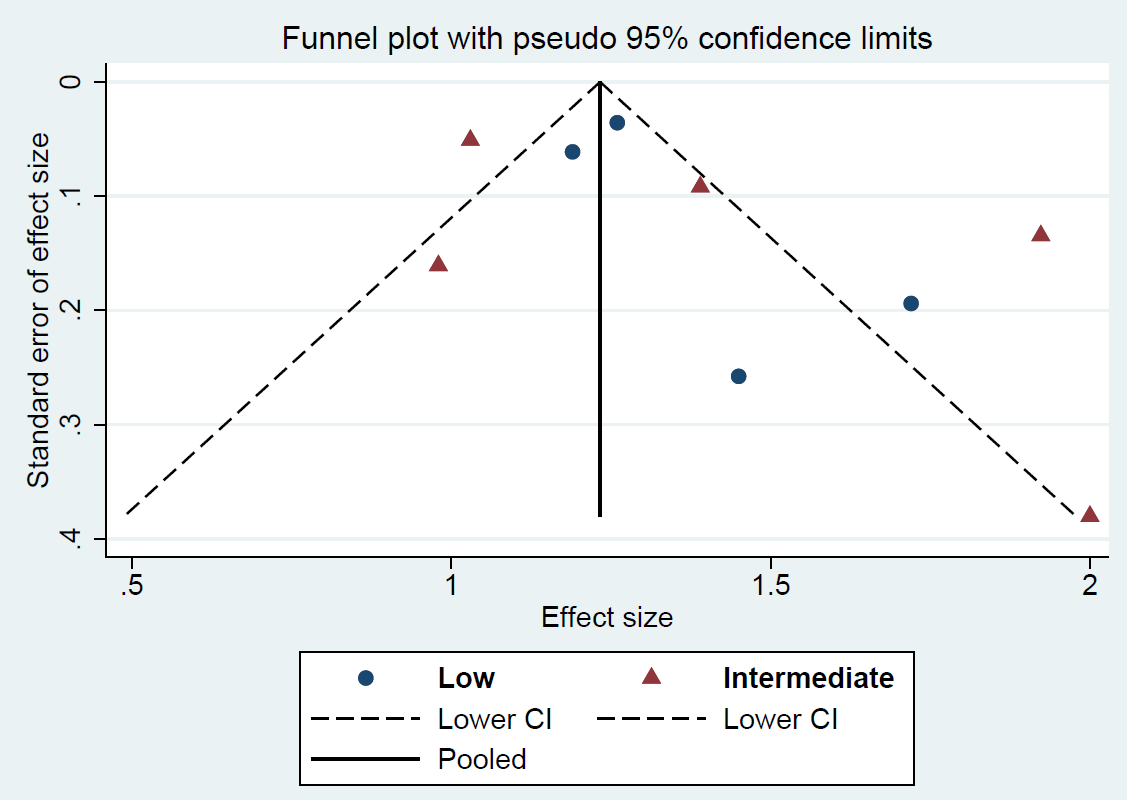


**Physical activity and no pollution**


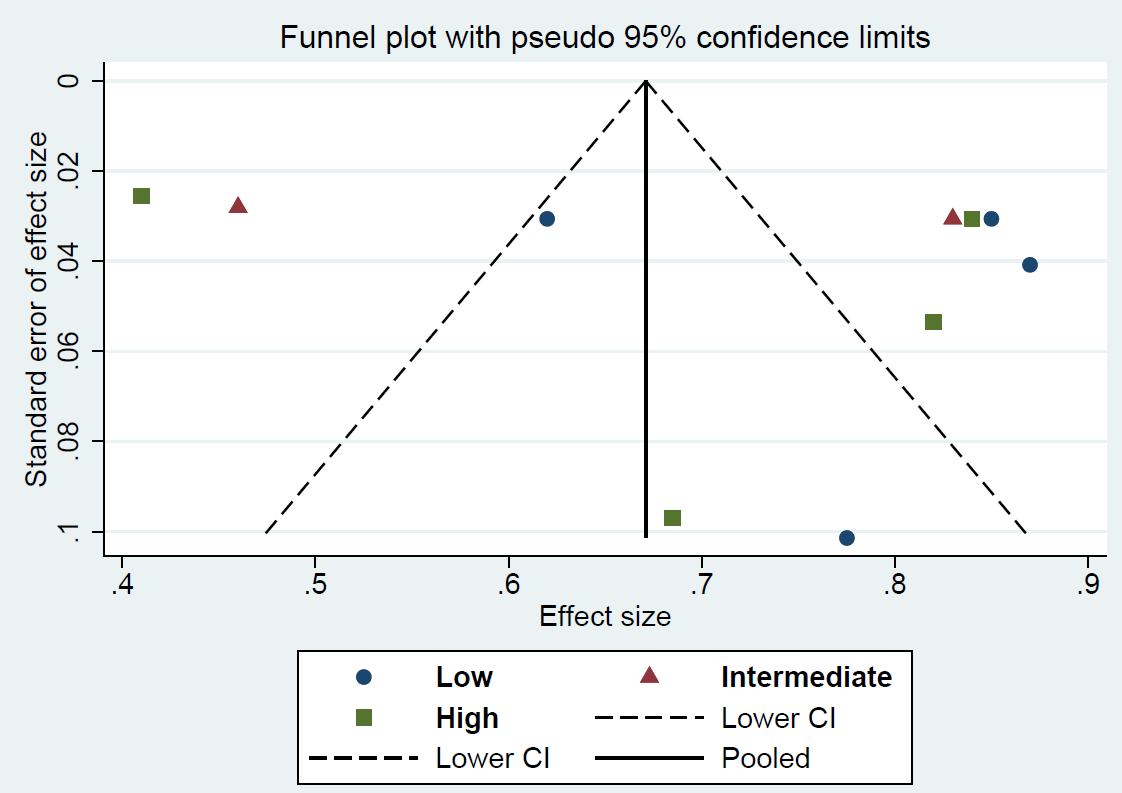


**Physical activity in high air pollution**


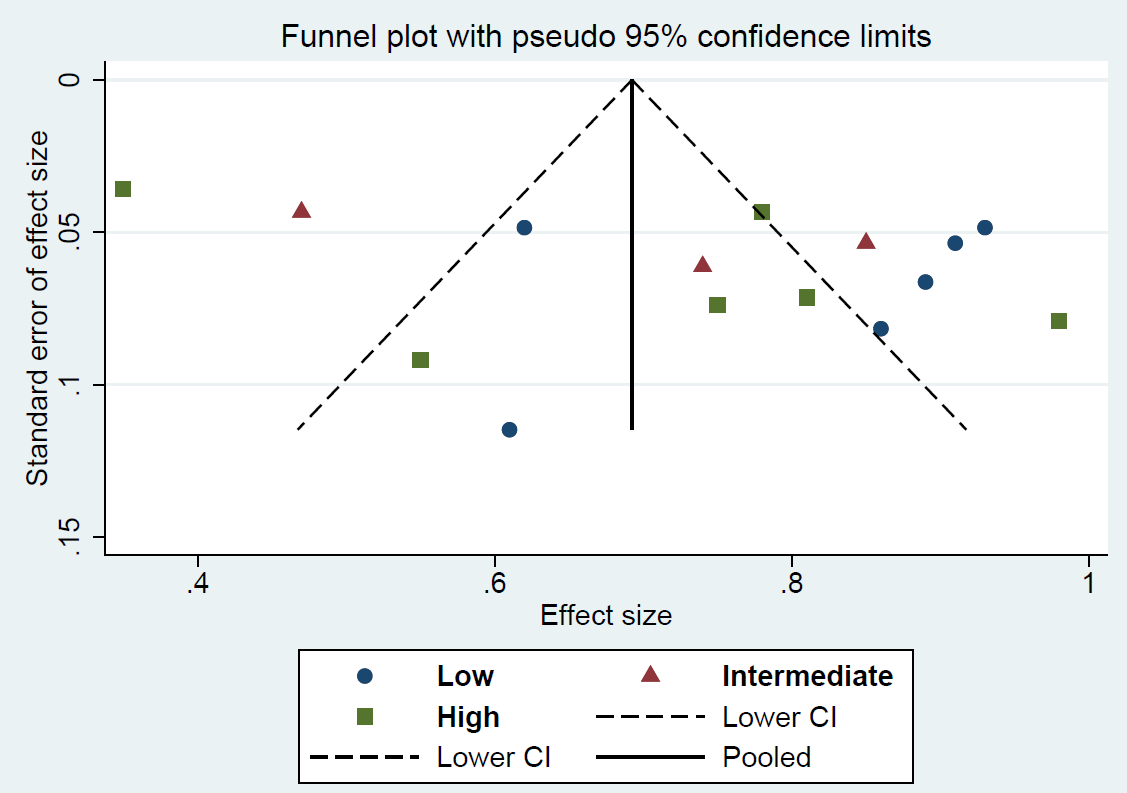

Supplement: Supplementary file 3 — Additional file 3. [file 40798_2025_830_MOESM3_ESM.docx]
