# Supplementary material for: Physical Activity, Air Pollution, and Mortality: A Systematic Review and Meta-analysis
Source: Sports Med Open. 2025 Apr 7;11:35. doi: 10.1186/s40798-025-00830-z (PMC11977067; doi:10.1186/s40798-025-00830-z)
Supplement: Supplementary file 4 — Additional file 4. [file 40798_2025_830_MOESM4_ESM.docx]

**Electronic supplementary material Table S1.** Details for the search strategy used within each database

**Pubmed**

(«air pollutants»[MH] OR «air pollutants»[TW] OR «air pollutant»[TW] OR «air pollution»[TW] OR «air pollution»[MH])

**AND** (exercise[MH] OR exercise[TW] OR exercises[TW] OR «physical activities»[TW] OR «physical activity»[TW] OR sports[MH] OR sport[TW] OR sports[TW])

**AND** (mortality[MH] OR mortality[TW] OR «mortalities»[TW] OR death[MH] OR «death rate» [TW] OR «death rates»[TW])

Filter Language = none

Filter Dates = none

**Cochrane Library**

("air pollutants" OR "air pollutant" OR "air pollution" )

**AND** ("exercise" OR "exercises" OR "physical activities" OR "physical activity" OR "sport" OR "sports" )

**AND** ( "mortality" OR "mortalities" OR "death rate" OR "death rates" )

Filter Language = none

Filter Dates = none

**ScienceDirect**

Pollution

**AND**

(exercise OR physical activity)

**AND**

mortality

Filter Language = english or french

Filter Dates = none

**Embase**

('air pollutants'/exp OR 'air pollutants' OR 'air pollutant'/exp OR 'air pollutant' OR 'air pollution'/exp OR 'air pollution')

**AND** ('exercise'/exp OR 'exercise' OR 'exercises' OR 'physical activities' OR 'physical activity'/exp OR 'physical activity' OR 'sport'/exp OR 'sport' OR 'sports'/exp OR 'sports')

**AND** ('mortality'/exp OR 'mortality' OR 'mortalities' OR 'death'/exp OR 'death rate'/exp OR 'death rate' OR 'death rates')

**AND** [embase]/lim NOT ([embase]/lim

**AND** [medline]/lim)

**AND** ('article'/it OR 'article in press'/it OR 'review'/it)

**AND** 'human'/de
