# Supplementary material for: Physical Activity, Air Pollution, and Mortality: A Systematic Review and Meta-analysis
Source: Sports Med Open. 2025 Apr 7;11:35. doi: 10.1186/s40798-025-00830-z (PMC11977067; doi:10.1186/s40798-025-00830-z)
Supplement: Supplementary file 5 — Additional file 5. [file 40798_2025_830_MOESM5_ESM.docx]

**Electronic supplementary material Table S2** Grading of Recommendation, Assessment, Development, and Evaluation (GRADE) instrument – Certainty of the evidence for our main outcome and for most secondary outcomes

| **Outcome** | **Study**  **design** | **n**  **studies** | **Certainty assessment** | | | |  | **Number**  **of patients** | **Effect OR**  **95% CI** | **Quality of evidence^1^** | **Importance of outcome** |  |
| --- | --- | --- | --- | --- | --- | --- | --- | --- | --- | --- | --- | --- |
|  |  |  | **Risk of**  **bias** | **Risk of Inconsistency** | **Risk of Indirectness** | **Risk of imprecision** |  |  |  |  |  |  |
| **Air pollution and no physical activity** | |  |  |  |  |  |  |  |  |  |  |  |
| Moderate air pollution | observational | 4 | low | significant | low | low |  | 1 299 859 | 1.29  (1.16 to 1.43 | ⨁⨁◯ ◯ | not important |  |
| High air pollution | observational | 5 | low | significant | low | moderate |  | 1 376 035 | 1.41  (1.05 to 1.77) | ⨁⨁◯ ◯ | not important |  |
| **Overall risk of mortality** | **observational** | **5** | **low** | **significant** | **low** | **low** |  | **1 376 035** | **1.36**  **(1,05 to 1.52)** | ⨁⨁⨁◯ | important |  |
| **Physical activity and no pollution** | |  |  |  |  |  |  |  |  |  |  |  |
| Low physical activity | observational | 4 | moderate | significant | low | low |  | 552 239 | 0.78  (0.64 to 0.92) | ⨁⨁◯ ◯ | not important |  |
| Moderate physical activity | observational | 2 | moderate | significant | low | moderate |  | 441 535 | 0.64  (0.28 to 1.01) | ⨁◯◯ ◯ | not important |  |
| High physical activity | observational | 4 | moderate | significant | low | low |  | 552 239 | 0.63  (0.33 to 0.94) | ⨁⨁◯ ◯ | not important |  |
| **Overall risk of mortality** | observational | 4 | moderate | **significant** | low | low |  | **552 239** | 0.69  (0.42 to 0.95) | ⨁⨁⨁◯ | important |  |
| **Physical activity in high air pollution** | |  |  |  |  |  |  |  |  |  |  |  |
| Low physical activity | observational | 6 | low | significant | low | low |  | 1 765 509 | 0.81  (0.69 to 0.93) | ⨁⨁⨁◯ | important |  |
| Moderate physical activity | observational | 3 | moderate | significant | low | moderate |  | 462 811 | 0.68  (0.44 to 0.93) | ⨁⨁◯ ◯ | important |  |
| High physical activity | observational | 6 | low | significant | low | low |  | 1 765 509 | 0.70  (0.49 to 0.91) | ⨁⨁⨁◯ | important |  |
| **Overall risk of mortality** | **observational** | **6** | **low** | **significant** | **low** | **low** |  | **1 765 509** | **0.74**  **(0.63 to 0.85)** | ⨁⨁⨁⨁ | **critical** |  |
| ^1^ We downgraded the quality of evidence of all outcomes when based on four or a smaller number of studies. | | | | | | | | | | | | |
